# Association between blood cholesterol and sodium intake in hypertensive women with excess weight

**DOI:** 10.1097/MD.0000000000010371

**Published:** 2018-04-13

**Authors:** Bruna Merten Padilha, Raphaela Costa Ferreira, Nassib Bezerra Bueno, Rafael Miranda Tassitano, Lidiana de Souza Holanda, Sandra Mary Lima Vasconcelos, Poliana Coelho Cabral

**Affiliations:** a Faculty of Nutrition, Federal University of Alagoas, Av. Lourival Melo Mota, Alagoas; b Department of Physical Education, Federal Rural University of Pernambuco, R. Dom Manoel de Medeiros; c Department of Nutrition, Federal University of Pernambuco, Av. Prof. Moraes Rego, Recife, Pernambuco, Brazil.

**Keywords:** anthropometry, cholesterol, hypertension, obesity, sodium

## Abstract

Restricted sodium intake has been recommended for more than 1 century for the treatment of hypertension. However, restriction seems to increase blood cholesterol. In women with excess weight, blood cholesterol may increase even more because of insulin resistance and the high lipolytic activity of adipose tissue.

The aim of this study was to assess the association between blood cholesterol and sodium intake in hypertensive women with and without excess weight.

This was a cross-sectional study with hypertensive and nondiabetic women aged 20 to 59 years, recruited at the primary healthcare units of Maceio, Alagoas, Brazilian Northeast. Excess weight was defined as body mass index (BMI) ≥25.0 kg/m^2^. Sodium intake was estimated by the 24-hour urinary excretion of sodium. Blood cholesterol was the primary outcome investigated by this study, and its relationship with sodium intake and other variables was assessed by Pearson correlation and multivariate linear regression using a significance level of 5%.

This study included 165 hypertensive women. Of these, 135 (81.8%) were with excess weight. The mean sodium intake was 3.7 g (±1.9) and 3.4 g (±2.4) in hypertensive women with and without excess weight, respectively. The multiple normal linear regression models fitted to the “blood cholesterol” in the 2 groups reveal that for the group of hypertensive women without excess weight only 1 independent variable “age” is statistically significant to explain the variability of the blood cholesterol levels. However, for the group of hypertensive women with excess weight, 2 independent variables, age and sodium intake, can statistically explain variations of the blood cholesterol levels.

Blood cholesterol is statistically inversely related to sodium intake for hypertensive women with excess weight, but it is not statistically related to sodium intake for hypertensive women without excess weight.

## 1 Introduction

A sodium intake of roughly 2.0 g/day^[1]^ to 2.3 g/day^[2]^ has been recommended for more than a century to treat hypertension and prevent cardiovascular and renal outcomes.^[3]^ This amount has been corroborated by a meta-analysis authored by He et al,^[4]^ who found that lower sodium intake significantly reduces blood pressure (BP) in hypertensive individuals regardless of gender and ethnicity.

However, these authors found that although lower sodium intake reduces BP without significantly affecting the levels of blood lipids (cholesterol triglycerides) and adrenaline, it makes some hormones slightly more active (renin, aldosterone, and noradrenaline).^[4]^

Likewise, the meta-analysis by Graudal et al^[5]^ also reported that low sodium intake reduces BP and increases renin, aldosterone and noradrenaline, but unlike He et al,^[4]^ they reported that lower sodium intake increases the levels of total cholesterol and triglycerides, leading to important complications and cardiovascular and endocrine implications.^[5]^

The abovementioned changes may be greater in hypertensive women with excess weight, especially abdominal adiposity, because obesity and hypertension are strongly related, whether as cause or coexisting factor. Different obesity-related mechanisms lead to an increase in BP, hemodynamic changes, impaired sodium homeostasis, renal dysfunction, autonomic nervous system imbalance, endocrine changes, oxidative stress, inflammation, and vascular lesion.^[6,7]^

Adipose tissue has high lipolytic activity, releasing fatty acids in the portal and systemic circulations. In the liver, fatty acids affect lipid metabolism and stimulate cholesterol synthesis, which, associated with the production of proinflammatory and proatherogenic cytokines, predisposes an individual to many metabolic and hemodynamic disorders.^[8]^

Given the above and the fact that 24-hour urine collection is considered the gold standard for estimating sodium intake,^[9]^ the present study aimed to assess the relationship between blood cholesterol and sodium intake in hypertensive women with and without excess weight.

## 2 Methods

This cross-sectional study, conducted from January to September 2015, included 165 hypertensive and nondiabetic women aged 20 to 59 years randomly recruited in primary healthcare units of the city of Maceio, capital of the state of Alagoas in the Brazilian Northeast. Pregnant women and women with genetic or acquired malformations were not included because anthropometric assessment would not be possible.

Since the present study is part of a research for the Unified Health System entitled “Consumption and food practices, modifiable risk factors for chronic diseases and hypertensive prognosis in the state of Alagoas,” whose sample size was estimated to identify the prevalence of risk factors in hypertensives, the study sample size was not estimated initially. However, the sample power (1-β) was calculated after data collection by the software Gpower 3.1 (Universität Düsseldorf, Dusseldorf, Germany) to assess the relationship between blood cholesterol and sodium intake in hypertensive women with excess weight, using a statistical significance level (α) of 0.05, and the resulting coefficient of determination and sample size.

Socioeconomic, demographic, anthropometric, body composition, biochemical, and BP data were collected. The self-reported races were grouped as white and nonwhite. Education level, based on full years of formal education, was classified as low (<8 years) and high (≥8 years). Socioeconomic status (SES) was determined by the Brazilian Criterion of Economic Classification, which attributes points to durable household items and family head's education level. The sum of these points classifies the population in the following economic classes: A1, A2, B1, B2, C1, C2, D, or E, in descending order, respectively, initiated by the highest purchasing income.^[10]^ Thus, SES was classified according to the following scheme: high SES (A1, A2, and B1), middle SES (B2 and C1), and low SES (C2, D, and E).

Anthropometric assessment consisted of measuring weight, height, and waist circumference (WC). The measurements were performed twice, by the same researcher, and averaged. New measurements were performed when the 2 weight, height, and WC measurements, differed by more than 100 g, 0.5 cm, and 0.3 cm, respectively.

Weight and height were measured as originally recommended by Lohman et al,^[11]^ using the electronic portable scale Lider model P200 M (maximum capacity of 200 kg and accuracy of 50 g) and the portable stadiometer Seca and its inelastic metric tape measure (length of 2 m, graduated to 0.1 cm). Body mass index (BMI) was given by dividing weight by the square of the height. Using the cut-off points provided by the World Health Organization (WHO),^[12]^ women with BMI below 25.0 kg/m^2^ were categorized as “without excess weight,” and those with BMI equal to or above 25.0 kg/m^2^, as “with excess weight.”

WC was measured by the inelastic metric fiberglass tape measure Sanny, with a length of 150 cm, graduated to 0.1 cm, at the midpoint between the lowest rib and the iliac crest at the end of a normal expiration.^[12]^ The waist-to-height ratio was calculated.

The percentage of body fat (BF) was calculated by the body composition assessment software CompCorp based on body resistance and reactance measured by the tetrapolar bioelectrical impedance device RJL model 101-A as recommended by Lukaski et al.^[13]^

Capillary blood was collected by disposable microcuvettes between 08:00 and 10:00 h after a 12-hour fast using Accu-Check (Roche) test strips and the portable monitor Accutrend GTC (Roche), which measures cholesterol in the 150 to 300 mg/dL range.

In order to determine sodium intake, the participants were asked to collect urine during a full 24-hour period, starting with the 2nd urine on day 1 and ending with the 1st urine on day 2. Urine was collected in a 5 L container provided by the researchers. When done, the participants returned the containers to their primary healthcare unit. The women were asked not to change their diets during the 24-hour urine collection period.

The analysis consisted of measuring urine volume using a 1000 mL beaker; collecting one 0.5 mL aliquot of urine using 1 mL pipettes and pipette pump; placing the aliquot in one 6 mL test tube; and measuring sodium concentration using the ion-selective electrode Iselab, with automatic aspiration, built-in printer, and automatic calibration and cleaning systems.

The urines of patients who failed to collect one or more urinations, containers with less than 500 mL of urine, and urine collected outside of the 23 to 25-hour period were discarded, as they are considered incomplete and/or inappropriate.^[14]^

The biochemical marker of sodium intake is the 24-hour urinary sodium excretion (24hUNaE) as more than 90% of the ingested sodium is excreted in the urine.^[9]^ The 24hUNaE is given by the formula: 24hUNaE (mmol/L) = 24-hour urine volume (mL) × excreted Na (mmol)/1000. 24hUNaE in mmol/L was converted into mg/L by multiplying it by sodium's molar mass (Na = 23 g).

BP was measured as recommended by the VII Joint National Committee of Hypertension^[15]^ by the automatic digital device Omron model HEM 705 CP, validated as instructed by international protocol.

The statistical analyses were performed by the software Epi Info version 7 (CDC/WHO, Atlanta, GE) and SPSS version 13.0 (SPSS Inc., Chicago, IL). The Kolmogorov–Smirnov test checked the normality of the continuous variables. Since these variables had normal distribution, they were expressed as mean and standard deviation. Student *t* test compared the means and Pearson correlation coefficient was used to assess the correlation coefficients. Bivariate analysis included all possible confounders. A multivariate linear regression model determined whether blood cholesterol was associated with sodium intake in hypertensive women with and without excess weight. The model includes the variables associated with the outcome in the bivariate analysis (*P* < .05). The beta coefficient was estimated. Variables with a significance level of 5% (*P* < .05) were considered significant for the final model.

This study was sponsored by a scientific research-funding agency owned by the state and complied with human research rules established by Resolution n. 466/2012 of the National Health Council. The study was approved in 2013 by the Research Ethics Committee of the Federal University of Alagoas (CAAE: 19203313.2.0000.5013). All women who agreed to participate in the study signed an informed consent form after being informed of the possibilities of risk and discomfort associated with the procedures.

## 3 Results

A total of 191 hypertensive women were assessed. Of these, 18 (9.4%) were excluded because their 24-hour urine collections were incomplete, and 8 (4.2%), because their blood cholesterol data were missing. Hence, the study included 165 hypertensive women, of which 138 (83.6%; 95% confidence interval [95%CI]: 76.9–88.7) were nonwhite, 122 (73.9%, 95%CI: 66.4–80.3) had less than 8 years of formal education, 127 (77%, 95%CI: 69.6–83.0) were low SES, and 135 (81.8%, 95%CI: 74.9–87.2) had excess weight (BMI ≥ 25.0 kg/m^2^). Their ages ranged from 28 to 59 years (48.7 ± 7.7).

Anthropometric and body composition variables were the only characteristics in the hypertensive women that varied significantly (Table 1).

**Table 1:**
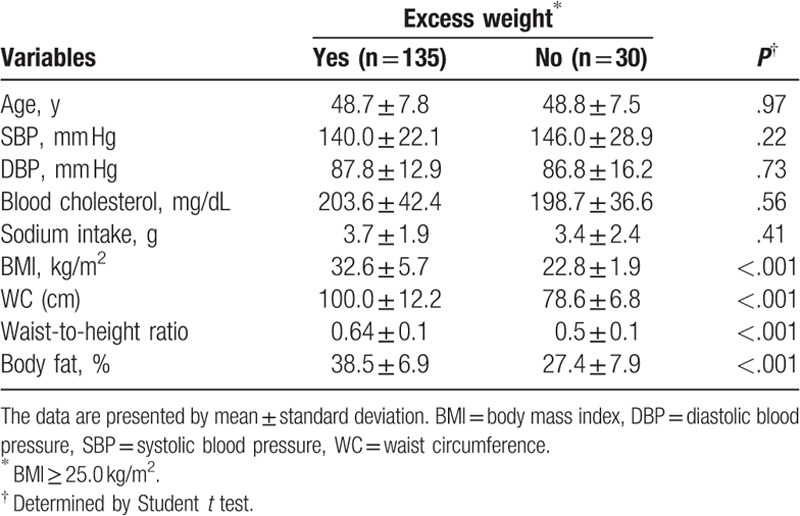
Characteristics of hypertensive women according to excess weight status.

Table 2 shows the correlation analyses between these variables, according to the excess weight status of the hypertensive women studied. These analyses show that blood cholesterol is negatively correlated with sodium intake (*r* = −0.167, *P* = .04) in women with excess weight, and positively correlated with age (*r* = 0.307, *P* < .001 vs *r* = 0.444, *P* = .01) in all study women.

**Table 2:**
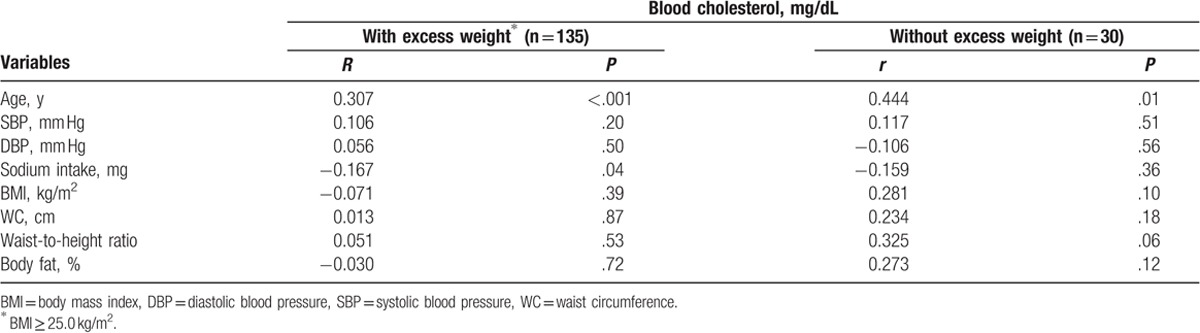
Pearson correlation coefficients (*r*) between blood cholesterol with age, blood pressure, sodium intake, and anthropometric and body composition measurements, according to the excess weight status of hypertensive women.

In order to explain the behavior of the dependent variable “blood cholesterol” (mg/dL) in the 2 groups of hypertensive (and nondiabetic) women with and without excess weight, the following independent variables are considered in the complete normal linear regression models: age (years), SBP (mm Hg), DBP (mm Hg), sodium intake (g), BMI (kg/m^2^), WC (cm), waist-to-height ratio, and BF (%). The 2 multiple normal linear regression models with these 8 independent variables are fitted to the blood cholesterol in the 2 groups. The results from these fits reveal that for the group of hypertensive women without excess weight only the independent variable “age” is statistically significant to explain the variability of the blood cholesterol. However, for the group of hypertensive women with excess weight, 2 independent variables, age and sodium intake, can statistically explain variations of the blood cholesterol levels. The significance of the independent variable is taken under a *P*-value less than 5% significance level.

Further, we perform the fits of the multiple normal linear reduced regression models in the 2 groups containing only the predictor variables which are statistically significant. The estimated coefficients, their 95%CI, and *P*-values are given in Table 3. For the group of hypertensive women with excess weight, we can conclude that an increasing of 1 g of sodium intake yields an average reduction of 3.517 mg/dL in the blood cholesterol, which corresponds to the estimated coefficient (=−3.517) of this independent variable. Further, hypertensive women with excess weight who are 10 years older have an average increase of 16.29 mg/dL in the blood cholesterol level. On the other hand, for hypertensive women without excess weight, an increase of 10 years in the age will produce an average increase of 18.99 mg/dL in the blood cholesterol.

**Table 3:**
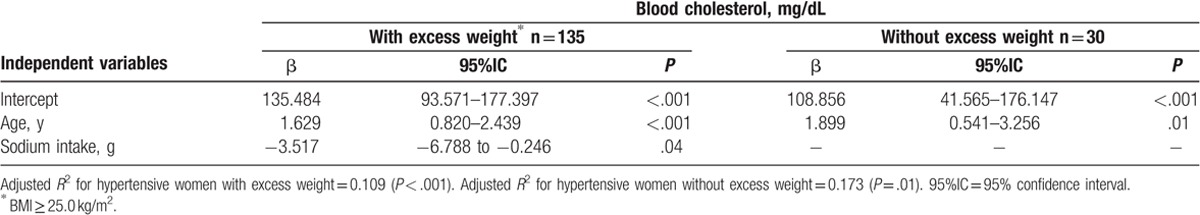
Multiple linear regression analysis of blood cholesterol in hypertensive women, according to excess weight status.

**Figure 1:**
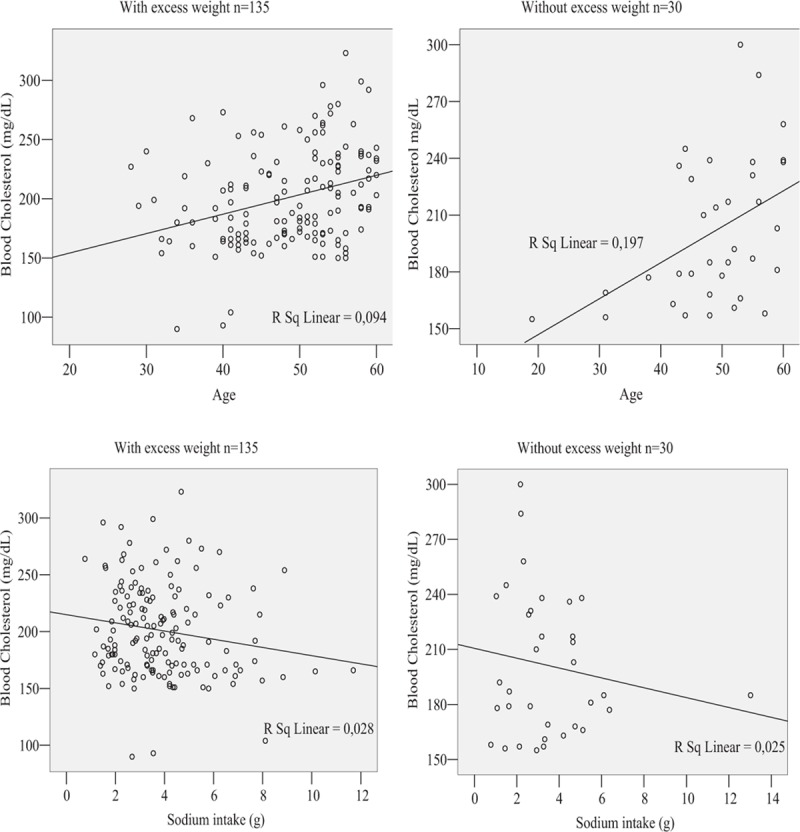
Scatter plots of association of age and sodium intake with blood cholesterol estimated from hypertensive women, according to excess weight status.

The estimated coefficients in Table 3 are in full agreement with the scatter plots displayed in Fig. 1 of the blood cholesterol versus age and sodium intake for the 2 groups of women. These plots really indicate a positive correlation between blood cholesterol and age and a negative correlation between blood cholesterol and sodium intake for the 2 groups, although the sodium intake does not explain statistically significant variation in the blood cholesterol of the hypertensive women without excess weight.

Based on the partial coefficient of determination of sodium intake on blood cholesterol obtained by multivariate regression analysis (partial *R*^2^ = 0.249), the sample had a power of 50% to detect a significant difference in this procedure.

## 4 Discussion

Blood cholesterol is inversely related to sodium intake in the hypertensive women with excess weight. Although their mean sodium intake was above the amount recommended by the WHO (2.0 g/day)^[1]^ and by the Institute of Medicine (2.3 g/day),^[2]^ the results were consistent with those of meta-analyses, which have evidenced that sodium restriction increases blood cholesterol.^[5,16–18]^

The mechanisms associated with the changes in these lipids seem to be related to the fact that limited sodium intake reduces body water content, and in an attempt to revert low plasma volume, epinephrine, renin, and angiotensin increase. These hormones inhibit insulin, causing insulin resistance^[18]^ and consequently, high insulin, compromising lipid metabolism, and increasing blood cholesterol.^[19]^

The relationship between epinephrine and insulin resistance is well known,^[20]^ and the interaction between the renin-angiotensin-aldosterone system and insulin resistance has been gaining increasing attention.^[21]^ Angiotensin seems to be the main underlying promotor of these effects as its AT_1_R receptor acts on the metabolism of glucose in the pancreas, adipose tissue, and skeletal muscle. In this last case, hemodynamic effects, such as vasoconstriction, which reduces local blood flow, and the direct effects on the insulin signaling pathway, which reduces glucose transporter type 4 (GLUT4) translocation to the cell membrane, strongly contribute to insulin resistance.

Given that hypertensive women with excess weight are susceptible to insulin resistance^[22]^ and that the study hypertensive women with excess weight had a mean BMI in the obesity range (BMI ≥ 30 kg/m^2^), mean WC in the high-risk range (WC ≥88 cm),^[12]^ and mean BF reasonably above the cut-off point associated with obesity (BF ≥ 32.0%),^[11]^ hypertension may have been exacerbated.

In addition to stimulating the synthesis of blood cholesterol in the liver,^[8]^ adipose tissue promotes inflammation and oxidative stress, which lead to insulin resistance and higher production of renin-angiotensin-aldosterone system components. Hence, adipose tissue has systemic effects on the regulation of BP and lipids.^[23]^ Also, the study sample consisted of mature hypertensive women, who are more susceptible to oxidative stress^[24]^ and consequently, to insulin resistance.^[19]^ All these aspects seem to clarify the inverse relationship between sodium intake and blood cholesterol in the study of hypertensive women with excess weight. Meanwhile, the positive relationship between age and blood cholesterol has been well documented.^[25,26]^

In a literature review, Graudal^[27]^ found that not one randomized clinical trial or observational study has supported a sodium intake below 2.645 mg, and that an intake above 4.945 mg is related to higher cardiovascular morbidity. Therefore, other studies are necessary to determine an optimal sodium intake, that is, one that controls BP in hypertensives without causing health-damaging metabolic changes.

Restricting sodium intake severely in a population with high habitual intake may promote low dietary adherence, poor diet, and nutritional deficiencies, which reinforce the need to further study sodium restriction in the treatment of hypertension.

The study strengths are: measured, not self-reported, anthropometric, biochemical, and BP data, and using the gold standard for sodium analysis. One limitation is the cross-sectional study design, which prevents identifying the order of occurrence of exposure and outcome, and thus, the establishment of causality in the study relationship. A 2nd limitation was the sample size, not large enough. Yet, it was still possible to find significant differences between hypertensive women with and without excess weight.

In conclusion, blood cholesterol is statistically inversely related to sodium intake for hypertensive women with excess weight, but it is not statistically related to sodium intake for hypertensive women without excess weight.

## Acknowledgments

The authors thank the Research Support Foundation of the state of Alagoas (Fundação de Amparo à Pesquisa do Estado de Alagoas) for sponsoring the study. The authors also thank Professor Gauss M. Cordeiro, PhD in Statistics from Imperial College (University of London) and professor at the Statistics Department of the Federal University of Pernambuco.

## Author contributions

**Conceptualization:** Poliana Coelho Cabral.

**Data curation:** Bruna Merten Padilha, Raphaela Costa Ferreira, Sandra Mary Lima Vasconcelos.

**Formal analysis:** Bruna Merten Padilha, Nassib Bezerra Bueno, Rafael Miranda Tassitano.

**Investigation:** Bruna Merten Padilha, Raphaela Costa Ferreira, Sandra Mary Lima Vasconcelos.

**Methodology:** Bruna Merten Padilha, Nassib Bezerra Bueno, Rafael Miranda Tassitano, Poliana Coelho Cabral.

**Project administration:** Sandra Mary Lima Vasconcelos.

**Software:** Nassib Bezerra Bueno, Rafael Miranda Tassitano.

**Supervision:** Sandra Mary Lima Vasconcelos, Poliana Coelho Cabral.

**Validation:** Poliana Coelho Cabral.

**Writing – original draft:** Bruna Merten Padilha.

**Writing – review & editing:** Raphaela Costa Ferreira, Lidiana De Souza Holanda, Poliana Coelho Cabral.
